# Natural statistics of human head orientation constrain models of vestibular processing

**DOI:** 10.21203/rs.3.rs-2412413/v1

**Published:** 2023-01-11

**Authors:** Christian Sinnott, Peter A. Hausamann, Paul R. MacNeilage

**Affiliations:** 1 University of Nevada, Department of Psychology, Reno, 89557, United States of America; 2 Technical University of Munich, Department of Electrical and Computer Engineering, Munich, 80333, Germany

## Abstract

Head orientation relative to gravity determines how gravity-dependent environmental structure is sampled by the visual system, as well as how gravity itself is sampled by the vestibular system. Therefore, both visual and vestibular sensory processing should be shaped by the statistics of head orientation relative to gravity. Here we report the statistics of human head orientation during unconstrained natural activities in humans for the first time, and we explore implications for models of vestibular processing. We find that the distribution of head pitch is more variable than head roll and that the head pitch distribution is asymmetrical with an over-representation of downward head pitch, consistent with ground-looking behavior. We further show that pitch and roll distributions can be used as empirical priors in a Bayesian framework to explain previously measured biases in perception of both roll and pitch. We also analyze the dynamics of human head orientation to better understand how gravitational and inertial acceleration are processed by the vestibular system. Gravitational acceleration dominates at low frequencies and inertial acceleration dominates at higher frequencies. The change in relative power of gravitational and inertial components as a function of frequency places empirical constraints on dynamic models of vestibular processing. We conclude with a discussion of methodological considerations and scientific and applied domains that will benefit from continued measurement and analysis of natural head movements moving forward.

## Introduction

Head orientation relative to gravity shapes statistical regularity of sensory stimulation across modalities^1^. More specifically, there is gravity-dependent structure in the sensory environment, and the orientation of the head relative to gravity determines how this structure is sampled by head-fixed sensory organs like the eyes, ears, and vestibular organs. Statistical regularity in sensory stimulation is therefore jointly mediated by regularity in head orientation and regularity in gravity-dependent environmental structure, and the combination of these two factors shapes sensory processing. At the encoding stage, more resources are devoted to encoding most common stimuli, and at the decoding stage, prior distributions across encountered stimuli shape how behaviorally and perceptually relevant states are estimated^2,3^. Thus, it is necessary to characterize statistical regularity in head orientation in order to better understand how this regularity shapes sensory encoding and decoding in the nervous system.

Prior research that comprehensively characterizes how the head is oriented relative to gravity during everyday activities is currently lacking in the literature. Among existing studies of natural head movement, the statistics of head orientation relative to gravity seems to be neglected^1^. Instead, previous work has characterized vestibular stimulation, that is the linear acceleration and angular velocity experienced by the head during natural tasks in humans^4,5^ as well as in non-human primates and rodents^6^. One very recent study does report statistics of head roll relative to gravity, but only during prescribed activities^7^. To our knowledge, no studies report statistics of head pitch relative to gravity.

One reason that head orientation relative to gravity is seldom reported is that it is difficult to measure during unconstrained natural behavior. Previous methods to track angular head position are either confined to the lab, or they are unable to provide robust positional measurement due to gravitoinertial ambiguity^1,8,9^. A recent solution which allows unconstrained, positional tracking is simultaneous localization and mapping (SLAM). SLAM and variants like visual-inertial simultaneous localization and mapping (VI-SLAM) fuse information from multiple sensors such as cameras and inertial measurement units (IMUs), allowing for robust positional tracking. SLAM and VI-SLAM have recently been used to track head movement during natural behaviors^9–11^. Here we use a commercial VI-SLAM solution, the Intel RealSense T265 (T265), which has been validated against an optical tracking volume to track human head movement^9^.

We use the T265 to measure statistics of natural head orientation during unconstrained natural activities over five hours of continuous recording. We then use these measures to evaluate a Bayesian model of vestibular sensory processing and perception^7,12–14^. In particular, we seek to explain observed, predominantly attractive biases in perception of head and body orientation^13,15^. Previous efforts to model these biases have used Bayesian frameworks where statistical regularities can influence perception via Bayesian priors. These priors are typically modeled as Gaussian, with the variability of the prior left as a free parameter^7,13^. In the current study we explore using empirical measures of the statistics of head pitch and roll to determine priors in the Bayesian model.

In addition, we address a longstanding question in the vestibular literature regarding tilt-translation processing. The otoliths respond in equivalent fashion to gravitational and inertial acceleration, and it is debated how the nervous system overcomes this ambiguity^16,17^. Central to some proposed methods are assumptions regarding the relative power of tilt and translation stimuli as a function of frequency^18,19^. For the first time, we evaluate relative power (i.e. power spectra) of tilt and translation components during natural everyday behavior and identify a crossing-point (a central parameter of several models) below and above which tilt and translation components have most power. Overall, this work demonstrates the value of measuring statistics of behavior and stimulation to constrain models of sensory processing.

## Methods

**Figure 1.**
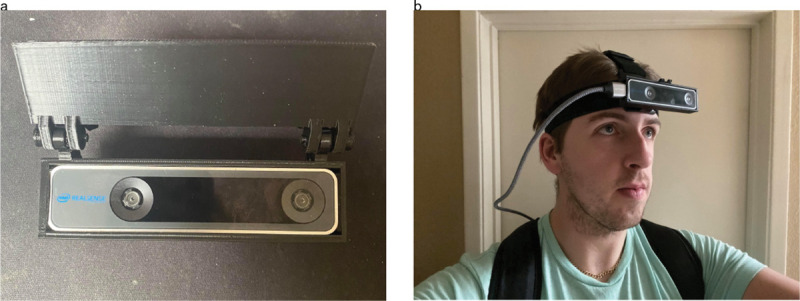
Recording equipment. The T265 (1a) measures 6DOF position of the camera relative to the world, and is worn on the head by a participant over five hours (1b). The camera plugs into a lightweight laptop worn in a backpack.

Ten participants (6 male, 3 female, 1 nonbinary identifying) were recruited to participate in this study. Data collection and all other procedures were approved by the Institutional Review Board at the University of Nevada, Reno and carried out in accordance with relevant guidelines and regulations. Researchers obtained separate, written informed consent from participants for use of participant likenesses or identifying images in figures in the current study (e.g. Fig. 1). This separate informed consent process was also approved by the Institutional Review Board at the University of Nevada, Reno. We asked participants to wear the T265 over a continuous five-hour recording period and to engage in natural behaviors that they were comfortable to have recorded. We also instructed participants to avoid activities that would compromise or be impeded by the recording equipment (e.g. high-impact sports), and to periodically re-calibrate the T265 every thirty minutes by slowly nodding and shaking their head five times (see Calibration subsection). All participants gave verbal informed consent in-line with data collection procedures approved by the Institutional Review Board at the University of Nevada, Reno.

## Hardware

Recording equipment consisted of the T265 tracking camera which was worn by participants on their head using a commercially available elastic strap harness (Fig. 1a). This off-the-shelf tracking camera uses VI-SLAM to estimate position of the camera relative to the world. It contains multiple sensors including two global shutter fisheye cameras, an accelerometer, and a gyroscope. The cameras sample video at 30 Hz, with an individual resolution of 848 × 800 pixels and a combined diagonal field of view of 173 degrees, while the gyroscope records at 200 Hz and the accelerometer at 62.5 Hz. The visual data from the cameras and the inertial data collected from the gyroscope and accelerometer are processed on an internal board with a proprietary VI-SLAM algorithm, yielding 6 degree of freedom (6DOF) odometry at 200 Hz. The current study uses odometry data down-sampled to 62.5 Hz, matching the sampling rate of the accelerometer to allow disambiguation of acceleration driven by gravity and self-motion. We also sub-sampled video at 1/60 Hz (1 frame per minute), in order to annotate activities for a separate experiment. This tracking camera has been validated for use in tracking human head movement^9^.

The T265 was connected via USB cable to a lightweight, portable laptop (Dell Latitude 5300). The laptop had an 8th generation Intel i7 CPU and 16 GB of DDR4 RAM. The laptop was carried by the participant in a backpack (Fig. 1b). Data acquisition software was programmed in Python 3.

## Calibration

To transform data from camera to head coordinates we measured the offset between the two reference frames at the beginning and end of recording. We first recorded a 15-second segment in which participants held their head in a static position such that Reid’s baseline, an anatomical reference line based on the external meatus of the ear and canthus of the eye, was perpendicular to the direction of gravity. Then, participants slowly nodded their head up-and-down five times (pitch movement) and shook their head left-to-right five times (yaw movement). In addition, participants performed periodic re-calibrations roughly every 30 minutes consisting of only the nodding and shaking segments. This was to measure and correct for any slippage of the device during the recording period.

The calibration movements are used to calculate the offset of camera and head reference frames. First, we solve for the rotation matrix that aligns pitch and yaw angular velocities measured during the calibration period with the horizontal and vertical axes, respectively. Next, we calculate the rotation about the pitch axis that best aligns the horizontal plane with a plane parallel to Reid’s baseline, where said plane is defined as the plane perpendicular to the average direction of gravity during the Reid’s baseline segment of the calibration. Data is ultimately expressed in this kinematically and anatomically-defined head-centered reference frame that is consistent across participants.

## Pre-Processing

We performed manual and automated pre-processing on our data prior to further analysis. This was done to remove artifacts driven by epochs where tracking failure occurred, to remove segments used for calibration, and to parse data into separate “low” and “high” velocity categories. Time points of the calibration segments at the beginning and end of the recording period were manually annotated. After the experiment, we visually inspected the angular and linear velocity time-series data to identify re-calibration segments that occurred approximately every 30 minutes as well as epochs with clear tracking failure.

Additionally, exclusion was conducted based on the confidence metric generated by the T265 during motion tracking. The metric ranges from 0 (poor) to 3 (good) and all positional data with a confidence of less than 3 was excluded from further analysis. Data excluded in this way often consisted of momentary tracking failures. Finally, we noticed loss of tracking can lead to artifacts with momentarily high linear velocity estimates. Therefore, all data frames with estimated linear velocity above a threshold of 3.05 meters per second were also excluded. This threshold was selected based on reported average jogging speed among a sample of young adults^20^.

Finally, data were parsed into separate low and high linear velocity categories. This was done in order to observe potential differences in head orientation during more stationary (e.g. during static standing, sitting, or lying positions) versus more mobile epochs (e.g. during locomotion). We chose a cutoff value of 0.75 m/s by calculating the linear velocity norm observed during a subset of selected stationary and walking epochs, and selecting the value that best split the difference between the typical value observed during stationary activities (approximately 0 m/s) and the typical value observed during walking (approximately 1–1.5 m/s). After all pre-processing, 41.95 hours of data were used for further processing. Of these 41.95 hours of data, 38.7 hours of data (92.25%) were designated as low velocity while 3.19 (7.75%) were designated as high velocity.

## Modeling

We used a simple, static Bayesian modeling framework to investigate how empirically observed distributions of head roll and pitch might shape biases in perception of pitch and roll^7,12–14^. Previous research has observed “attractive” bias for head roll and pitch perception at most eccentricities^13,15^. This bias is termed “attractive” in a Bayesian framework, because perceptual estimates are attracted towards the mean of the prior distribution which is assumed to have maximal probability at upright. Functionally, this attractive bias towards the prior results in perceptual underestimation relative to the true stimulus value.

To generate model predictions of head roll and pitch perception bias, we generate kernel density estimates (KDEs) from distributions of head roll and pitch orientations across all participants. These are used as prior probability distributions in our Bayesian model, and multiplied with likelihood distributions at every whole degree value in a range of +/− 120° for head roll and +/−90° for head pitch. These ranges are selected because they correspond to ranges of pitch and roll perception measured in previous psychophysical research^13,15^, and we compare our model prediction with these results. Noise is applied to the likelihood functions as eccentricity increases either linearly (linear model) or non-linearly (shear model). The parameter that determines this multiplicative noise on the likelihood is the only free parameter of the model. The mean of the resultant posterior distribution is used as a point estimate for perception of a given roll or pitch angle. The value for the single free parameter that yielded the best-fitting model in each case was found by minimizing the residual standard error (RSE):

(1) $$RSE=\sqrt{\frac{1}{n\;-\;2}\sum_{i=1}^{n}{\left(y_{i}-{\hat{y}}_{1}\right)}^{2}}$$

For greater detail, see Supplemental Materials.

## Results

### Descriptive Statistics

**Figure 2.**
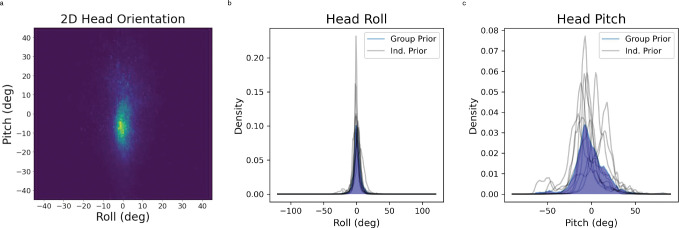
2D head orientation measured across all participants (2a). Marginal KDEs for roll (2b) and pitch (2c) are also plotted. The KDEs plotted in blue represent the distributions across all participants, while black traces represent KDEs for individual participants.

While roll and pitch are circular variables, neither roll nor pitch distributions were significantly wrapped (i.e. upside-down orientations were not observed), so we opted to use linear rather than circular descriptive statistics. Head roll was centered close to zero and had lower variability (M = 0.5806°, SD = 6.2108°). Head pitch was biased downward and had relatively high variability (M = −1.7701°, SD = 16.8167°) (Fig. 2a). To more completely characterize these distributions, we also calculated higher moments, including skewness and excess kurtosis, using the stats.describe function in the Python 3 library scipy (see Supplemental Material for formulae). Both roll (*μ*_3_ = 0.1211) and pitch (*μ*_3_ = −0.009) distributions had little skewness when measured in this way. Finally, the roll distribution had much greater excess kurtosis (*μ*_4_ = 7.2592) than the pitch distribution (*μ*_4_ = 1.7467).

We also calculate separate KDEs for head roll (Fig. 2b) and pitch (Fig. 2c) during low-velocity (<.75 m/s) and high-velocity (≥.75 m/s) epochs, which can be found in the supplemental materials. Generally, high-velocity head roll and pitch KDEs had decreased excess kurtosis relative to that observed in KDEs generated from data across all velocities. The high-velocity head pitch KDE also showed increased skewness and bias relative to the KDE generated from head pitch data across all velocities. Low-velocity head pitch and roll KDEs differed very little from their analogues generated from data of all velocities, as low-velocity data made up the majority of the total dataset (92.25% of all data). Greater details can be found in the supplemental materials.

### Power Spectra

**Figure 3.**
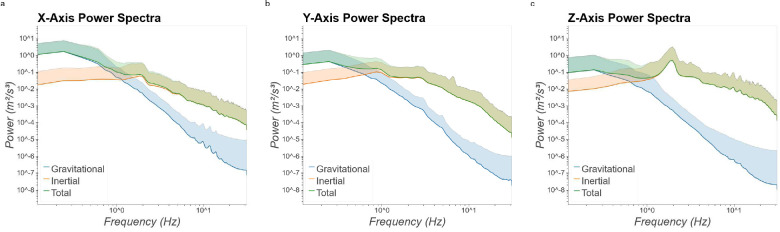
Power spectra for gravitational (blue), inertial (orange), and sum total (green) linear acceleration along X- (nasal-occipital), Y- (interaural), and Z- (dorsal-ventral) axes. Figure axes are log-scaled. Crossing points in power between gravitational and inertial acceleration are observed at 1.14, 0.625, and 0.635 Hz for X-, Y-, and Z-axes, respectively

Power spectra of measured linear acceleration were calculated in order to compare relative power of the gravitational and inertial components as a function of frequency. Ppower was calculated using the scipy.stats library in Python 3, with a sampling frequency (fs) of 62.5 and a fast Fourier transform length (nfft) of 512 samples. Along each axis, a crossing point was observed (Fig. 3). Below this critical frequency, gravitational acceleration exhibited the majority of power while above this frequency inertial acceleration exhibited greater power. These crossing points were observed at 1.14, 0.625, and 0.635 Hz for linear acceleration along the X- (nasal-occipital) (Fig. 3a), Y- (interaural) (Fig. 3b), and Z- (dorsal-ventral) (Fig. 3c) axes, respectively. We conducted similar analyses separately for low- and high-velocity epochs (see Supplementary Materials). Generally, crossing points were at higher frequencies across all axes for low-velocity epochs relative to all velocity epochs, while they were at slightly lower frequencies during high-velocity epochs. Furthermore, we observe a dominant peak at 2 Hz during high-velocity epoch along the Z-axis, likely driven by preferred stepping frequency during locomotion.

### Fitting Psychophysical Data

Bayesian models are fit to psychophysical data by adjusting the value of one free parameter (σ), which represents the multiplicative noise on the likelihood. This multiplicative noise varies as either a linear (linear noise model) or sinusoidal (shear noise model) function of the presented tilt angle. We determined the value that provided the best model fit by selecting the sigma value that minimizes distance (RSE) between the predicted and observed error.

**Figure 4.**
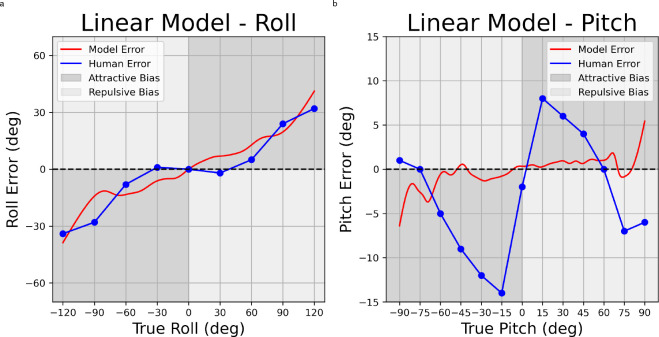
Psychophysical data (blue) and predictions of the linear noise models (red) for roll (4a) and pitch (4b). The presented stimulus orientation is plotted on the X-axis and estimation error (bias) is plotted on the Y-axis. Ticks and gridlines on the X-axis represent angles for which psychophysical data (blue) were previously reported: roll data is replotted from^13^, pitch data is replotted from^15^. Data between gridlines are linearly interpolated.

**Figure 5.**
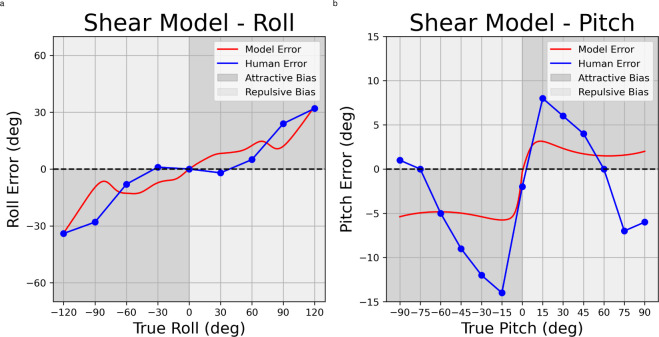
Utricular shear models for roll (5a) and pitch (5b). X- and Y- axes represent true orientation eccentricity and orientation eccentricity error, respectively. X-axis ticks and gridlines represent eccentricities where psychophysical data were reported in previous data, while psychophysical data between gridlines are linearly interpolated.

For roll perception, we compare model predictions to data from^13^ and we achieve a fit with 6.435 RSE (with σ = 0.113) for the linear noise model (Fig. 4a), and 8.344 RSE (with σ = 0.013) for the shear noise model (Fig. 5a). For pitch perception, we compare model predictions to data from^15^ and we achieve a fit with 6.779 RSE (with σ = 0.001) for the linear noise model (Fig. 4b), and 4.899 RSE (with σ = 0.132) for the shear noise model (Fig. 5b).

## Discussion

In the present study we measured head orientation relative to gravity during natural, everyday behaviors of ten participants over approximately five hours each. The aim was to characterize distributions of head orientation and relate these via Bayesian modeling to known biases in perception of upright. Below we first discuss the nature of these distributions, then we discuss the outcome of modeling efforts, and finally we discuss the dynamics of head orientation and its relation to overall linear acceleration measured at the head. We finish by reviewing certain methodological considerations as well as future research directions.

### Characteristics of Head Orientation Distributions

Cumulative across-subject pitch and roll distributions were generally centered near upright. For individual subjects, there was considerable variability in the location of the peak for pitch (grey lines, Fig. 2c). This could reflect calibration variability, differences in activities that were sampled for each participant, or individual differences in biomechanics. We suspect individual biomechanical differences are mostly responsible because most datasets included a significant amount of stationary activity and we expect calibration noise was minimal (see Methodological Considerations). Peaks for individual roll distributions, on the other hand, exhibited much less variability across all subjects.

In terms of variability, the cumulative pitch distribution was much more variable than roll. This reflects biomechanical differences in these dimensions, namely greater range of head-on-body flexion and extension as well as full-body flexion and extension for pitch relative to roll. Additionally there is a greater prevalence of natural variations in pitch during natural locomotion and orienting behaviors. In particular, previous work has robustly demonstrated the need for humans to gaze downwards when walking in order to find stable future footholds and prevent falling or injury, particularly in challenging environments that place high demands on postural control during locomotion^21–23^. To maintain downward gaze on footholds in the lower visual field, it follows that a persistent downward head pitch is needed. The high-velocity head pitch KDE, which captures primarily locomotion and other movement-based behaviors, supports this notion and shows increased downward bias and asymmetry relative to the low-velocity KDE generated from predominantly stationary behaviors.

This persistent downward head pitch is captured in the shape of the cumulative pitch distribution, which appears more asymmetrical than the roll distribution. This asymmetry is not captured by the standard skewness metric, but it has a significant impact on modeling results discussed below. We therefore further quantify this asymmetry as the ratio of variability for pitch that is backward compared to forward relative to the peak. This calculation yields an asymmetry ratio of 1.38, meaning that there was 38 percent greater variability in forward compared to backward pitch angles. The pitch and roll distributions also differed in their kurtosis. The roll distribution exhibited greater excess kurtosis than the pitch distribution, meaning that the roll distribution has relatively long tails; extreme roll values were observed, but these observations were relatively infrequent. This has implications for the modeling results discussed below.

### Fitting Psychophysical Data

Perception of upright has previously been modeled in a Bayesian framework with a prior distribution for head-in-space orientation that is centered at a value of zero tilt relative to gravity^12–14,24^. In this previous work, prior and likelihood are typically modeled as Gaussian distributions with parameters determining variability left free. Here, we instead use empirically-measured distributions as priors, an approach that enables more rigorous testing of the Bayesian framework (e.g.^25^); it allows for non-Gaussian priors and eliminates prior variability as a free parameter.

We fit two versions of this model, one in which variability increases linearly with increasing tilt angle and one in which it increases with the sine of the angle, i.e. proportional to the shear force at the utricle^26,27^. The multiplicative factor for increasing variability on the likelihood is the only free parameter of these models. The additive factor is taken from previous work in head roll modeling^13^. The same additive factor was used for head pitch modeling because perception of head pitch has not been modeled previously in this manner.

For roll perception, modeled biases increase with increasing roll angle, but exhibit a different trend than the measured bias. The measured bias remains near zero out to roughly 30°, then increases rapidly out to 90°; a trend known as the E-effect^28^. In contrast, the modeled bias (with both linear and non-linear noise) increases in a manner that is roughly constant with increasing tilt angle. Other models with Gaussian priors have included additional free parameters, such as degree of uncompensated ocular torsion, which allow these models to better capture the detailed shape of the perceived bias curve (e.g.^7,14^). Nevertheless, a simple Bayesian model with an empirical prior for upright provides a reasonable qualitative fit with only a single free parameter.

In a recent study similar to ours, Willemsen et al. also measure the empirical roll prior and explore a greater range of model variations with several additional free parameters^7^. They obtain the best fit with a Gaussian rather than an empirical prior. They note that the empirical prior leads to posterior probability distributions and thus perceptual estimates with variability that is actually increased relative to the likelihood, and that this is due to the excess kurtosis in the empirical prior compared to the Gaussian. The implication is that the nervous system may use a Gaussian approximation of an empirical prior to allow less variable roll estimates at more extreme angles. This intriguing finding does not detract from the importance of measuring empirical priors, but it does raise followup questions about the nature of this hypothesized approximation process.

For pitch perception, the linear noise model struggles to capture the detailed shape of the perceptual bias curve. This is because this model tends to predict attractive biases (i.e. underestimation) that increase with increasing tilt angle, as observed for roll. Pitch biases, in contrast, increase sharply out to +/− 15°, then decrease again, becoming repulsive (i.e. overestimation) at angles greater than 60° (Fig. 4b). Consequently, a least-square fit using the linear noise models settles on a multiplicative factor that predict little bias. The least-square fit achieved with the non-linear noise model, on the other hand, is able to capture the pattern of increasing bias out to +/− 15°, with bias that decreases beyond this point. This pattern can be traced back to to the non-linearity, that is the decreasing change in variability on the likelihood as the pitch angle increases, and how this interacts with the non-Gaussian empirical prior.

While imperfect, the non-linear noise model for pitch also captures another distinctive feature of the perceptual bias, that is asymmetrical bias magnitude that is considerably larger for backward compared to forward pitch; the maximum bias for backward pitch is 1.82 times the maximum for forward bias. This is a consequence of the asymmetrical pitch prior which exhibits variability that is 1.38 times greater for forward compared to backward pitch, thus accounting for the difference in bias amplitude. This qualitative match again supports the idea that perceptual biases are to some degree shaped by the natural statistics of human head orientation.

If perceptual bias is indeed shaped by stimulus statistics, one would expect differences in statistics across individuals to result in differences in individual biases as well. In future work, it would be possible to test this hypothesis for pitch perception specifically, for example by measuring asymmetry of an individual’s natural pitch distribution and testing how well this asymmetry predicts asymmetry in perceptual biases for that individual. Such a relationship would be particularly important to investigate in a clinical setting where biomechanical constraints associated with certain disorders could shape stimulus statistics and thus manifest as differences in perception of upright and perhaps even balance performance and fall risk.

### Power Spectra and Processing of Dynamic Vestibular Stimulation

While the dynamics of total linear acceleration have been reported previously, the separate dynamics of gravitational and inertial components during natural everyday activities over long time periods in humans have not been reported previously. As expected, we see acceleration due to gravity contribute more to the total power at lower frequencies and power due to inertial acceleration dominate at higher frequencies. Additionally, we see a peak in power at approximately 2 Hz along the Z-axis, corresponding well with previous work that demonstrates a strong peak of linear acceleration of the head along the vertical axis with 2 Hz stepping frequency^29^.

Power spectra of gravitational and inertial components of otolith stimulation are relevant for processing of ambiguous otolith stimulation, known as the tilt-translation ambiguity. An early suggestion for solving this ambiguity is frequency segregation, whereby low and high frequency otolith stimulation are interpreted as due to tilt and translation, respectively^30^. This simple heuristic approach has been successful in modeling otolith-ocular responses in both squirrel monkeys^18^ and humans^19^, with estimated cutoff frequencies 0.5 and 0.3 Hz, respectively. Later studies confirmed that frequency segregation could explain ocular responses in humans, but with a significantly lower cutoff frequency of 0.07 Hz^31,32^.

Observed crossing points in the power spectra (Fig. 3) where power transitions from predominantly gravitational to predominantly inertial acceleration provide empirical data to constrain selection of cutoff frequencies for frequency segregation models. We observe crossing points at frequencies that are higher than the values suggested by previous studies. Across the data set as a whole, these values are 1.14, 0.625, and 0.635 Hz for X-, Y-, and Z-axes, respectively. However, if we consider only high-velocity epochs these values fall to 1.101, 0.566, and 0.525 Hz for X, Y, and Z-axes, respectively (see Supplemental Material), which is more in line with previous research.

For resolving tilt-translation ambiguity, an alternative to frequency segregation is multi-modal (i.e. canal-otolith) integration^16^. While previous research has reported that reflexive eye movements can be explained by frequency segregation, perception was best explained based on internal models of canal-otolith interactions^31,32^. Recent models of canal-otolith interaction for perception have found that sensory integration is most crucial in the range of 0.2 to 0.5 Hz^33^. We note that the differing natural dynamics of gravitational and inertial components also have a role in shaping the performance of such statistically optimal multi-modal models.

On the one hand, noise characteristics of afferent sensory signals have recently been shown to be related to natural statistics of angular velocity stimulation for angular velocity^34^, and should thus influence measurement noise in the Kalman filter framework. The same should hold true for afferent linear acceleration signals and their relation to natural statistics of linear acceleration; the principle may also extend to neural populations that respond selectively to tilt and translation^35,36^. On the other hand, the distributions of input motion should also shape the modeled process noise of the Kalman filter. Ultimately, the Kalman gain, and thus the performance of such statistically optimal models as a whole, depends on the ratio of process to measurement noise which in turn depends on natural stimulus dynamics^37^. Further research should consider how best to constrain such models with measurements of natural head movement statistics.

### Methodological Considerations

Several recent studies have reported statistics of natural head movements, and it is therefore important to note differences across studies in terms of recording methods, coordinate frame conventions, and sampling procedures. In the current study, we use a commercially available VI-SLAM system which we have previously validated against an optical tracking system^9^. This method overcomes gravitoinertial ambiguity inherent in data reported by several previous studies^4,5^. The ambiguity inherent in IMUs data may be overcome using an IMUs with magnetometer and filtering methods^7^, but the accuracy of tilt and translation estimates derived from filtering should be validated in some way^8^.

In the present study, we report data in an anatomical reference frame, i.e. relative to Reid’s baseline, which we define as the line running from the canthus of the eye through the middle of the meatus of the ear. This is similar to, but not necessarily identical to, the line defined by the Frankfurt (or Frankfort) plane used as a reference in previous studies^4,5^, which runs through the bottom of the orbit and through the upper point of auditory canal. The alignment of data to this reference plane or line is subject to error because it is based on approximate visual alignment by the experimenter of either the sensor on the head or the head relative to gravity (current study). An alternative method would be to adopt a purely kinematic reference frame defined only by the two planes traced when the subject naturally shakes and nods the head, the third plane being identified as the one perpendicular to the intersection of the first two. This coordinate frame would not rely on identifying Reid’s baseline and would therefore have the advantage of avoiding errors introduced by approximate visual alignment and may be a more natural choice when studying head movement.

Finally, it is worth noting differences across studies in terms of what types of activities are sampled. The current study measured unprescribed activity over a longer time period; to our knowledge, this is the only study to report statistics of head orientation during unprescribed activities of daily life. Previous studies measuring unprescribed activities have reported head movements, as captured by an IMU, but not orientation^8,29^. Others report head movement and/or orientation during prescribed activities such as walking, climbing/descending stairs, running, and hopping^5,7^. Dynamic activities are likely over-represented in such studies and this likely impacts the reported statistics. It remains an open question as to what type of sampling is best-suited to which type of scientific inquiry. One advantage of long-term, unprescribed sampling is that it is more likely to be representative of natural behavior, and data can always be partitioned post-hoc based on approximate human activity recognition (see Supplemental Materials). This reveals that difference across behavioral modes can be significant and raises the possibility of mode- or activity dependent (e.g. locomotion-dependent) neural processing^38^.

### Conclusion

Here we report for the first time the statistics of head orientation relative to gravity as well as power spectra of naturally-experienced gravitational and inertial acceleration during everyday activity in humans. We show that these measures have implications for perception of head orientation, as well as processing of dynamic vestibular stimulation. Measures of head orientation are more broadly relevant because they not only constrain models of spatial orientation and vestibular processing, but also determine how the nervous system samples gravity-dependent visual and auditory structure in the environment. Future work is needed to investigate how these measures vary across different groups, such as children and clinical populations, across activities, as well as across species^1^. It is also interesting to consider how statistical measures such as these can inform more technological endeavors, such as predictive methods for head or gaze tracking that are relevant for emerging VR and AR technologies^39^. We expect that increased availability of head tracking data in the future will contribute to advances across these scientific and application domains.

## Data Availability

The dataset used and/or analysed during the current study is available from the corresponding author on reasonable request.
